# *TYK2* Promoter Variant Is Associated with Impaired Insulin Secretion and Lower Insulin Resistance in Japanese Type 2 Diabetes Patients

**DOI:** 10.3390/genes12030400

**Published:** 2021-03-11

**Authors:** Hitoe Mori, Hirokazu Takahashi, Keiichiro Mine, Ken Higashimoto, Kanako Inoue, Motoyasu Kojima, Shigetaka Kuroki, Takahisa Eguchi, Yasuhiro Ono, Sadataka Inuzuka, Hidenobu Soejima, Seiho Nagafuchi, Keizo Anzai

**Affiliations:** 1 Division of Metabolism and Endocrinology, Faculty of Medicine, Saga University, Saga 849-8501, Japan; 16624022@edu.cc.saga-u.ac.jp (H.M.); sv7899@cc.saga-u.ac.jp (K.M.); ki.0802@hotmail.co.jp (K.I.); mkojima.saga@gmail.com (M.K.); su2733@cc.saga-u.ac.jp (S.N.); akeizo@cc.saga-u.ac.jp (K.A.); 2 Liver Center, Faculty of Medicine, Saga University Hospital, Saga University, Saga 849-8501, Japan; 3 Division of Host Defense, Medical Institute of Bioregulation, Kyushu University, Fukuoka 812-8582, Japan; 4 Divison of Molecular Genetics & Epigenetics, Department of Biomolecular Sciences, Faculty of Medicine, Saga 849-8501, Japan; higashim@cc.saga-u.ac.jp (K.H.); soejimah@cc.saga-u.ac.jp (H.S.); 5 Saiseikai Karatsu Hospital, Saga 847-0852, Japan; 6 Eguchi Hospital, Saga 845-0032, Japan; kurokiskkuroki@yahoo.co.jp (S.K.); egumedica@yahoo.co.jp (T.E.); 7 Department of Internal Medicine, Kouhokai Takagi Hospital, Fukuoka 831-0016, Japan; ono@kouhoukai.org; 8 Inuzuka Hospital, Saga 849-1311, Japan; sadataka@inuzuka-hp.or.jp

## Abstract

Accumulating evidence has suggested that viral infection causes type 1 diabetes due to direct β-cell damage and the triggering of autoimmune reactivity to β cells. Here, we elucidated that the tyrosine kinase 2 *(Tyk2*) gene, encoding an interferon receptor signaling molecule, is responsible for virus-induced diabetes in mice, and its promoter variant confers a risk of type 1 diabetes in humans. This study investigated the relationship between a *TYK2* promoter variant *(TYK2PV)* and insulin secretion in type 2 diabetes patients. *TYK2PV* status was determined using direct DNA sequencing and its associations with fasting insulin, C-peptide, and homeostatic model assessment of insulin resistance (HOMA-IR) were evaluated in type 2 diabetes patients without sulfonylurea or insulin medication. Of the 172 patients assessed, 18 (10.5%) showed *TYK2PV*-positivity. Their body mass index (BMI) was significantly lower than in those without the variant (23.4 vs. 25.4 kg/m^2^, *p* = 0.025). Fasting insulin (3.9 vs. 6.2 μIU/mL, *p* = 0.007), C-peptide (1.37 vs. 1.76 ng/mL, *p* = 0.008), and HOMA-IR (1.39 vs. 2.05, *p* = 0.006) were lower in those with than in those without the variant. Multivariable analysis identified that *TYK2PV* was associated with fasting insulin ≤ 5 μIU/mL (odds ratio (OR) 3.63, *p* = 0.025) and C-peptide ≤ 1.0 ng/mL (OR 3.61, *p* = 0.028), and also lower insulin resistance (HOMA-IR ≤ 2.5; OR 8.60, *p* = 0.042). *TYK2PV* is associated with impaired insulin secretion and low insulin resistance in type 2 diabetes. Type 2 diabetes patients with *TYK2PV* should be carefully followed in order to receive the appropriate treatment including insulin injections.

## 1. Introduction

The prevalence of diabetes is increasing globally. The International Diabetes Federation reported that the number of diabetes cases reached 425 million in 2017, and this is predicted to total 693 million globally by 2045 [1]. Environmental factors including toxins and viruses, as well as changes in lifestyle, are considered to be associated with the increasing prevalence of diabetes [2]. Viral infection is well recognized as an etiological factor in type 1 diabetes. Both DNA and RNA viruses cause type 1 diabetes. In particular, enterovirus has been well studied in the context of the pathogenesis of type 1 diabetes [3,4]. Several previous epidemiological studies identified that an increased titer of blood enterovirus antibody is associated with the risk of type 1 diabetes. Circulating RNA of enterovirus has also been identified in the serum of patients with acute-onset type 1 diabetes [5]. Furthermore, it was reported that the expression of enterovirus antigen was observed in islet cells after the onset of type 1 diabetes [6,7,8]. These reports suggest that enterovirus infection is associated with the onset of type 1 diabetes.

Innate immunity is involved in the protection against viral infection. For example, pattern recognition receptors such as toll-like receptors and RIG-I-like (retinoic acid inducible gene-I) receptors on the surface of cells recognize the virus and increase the production of interferon (IFN) α/β via the activation of transcription factors [9]. IFNα/β binds to the IFN receptors (IFNR1, IFNR2) and activates the downstream signaling including the JAK/STAT pathway [10]. IFN and JAK/STAT signaling also play important roles in adaptive immunity. For example, IFNα/β induces the expression of co-stimulatory molecules such as CD40, CD80, CD86, and MHC, promoting the maturation of dendritic cells. It also induces chemokines and the migration of lymphocytes and monocytes to the site of inflammation. IFNα/β is also involved in the activation and proliferation of CD8^+^ T cells and the maintenance of memory CD8^+^ cells. In addition, IFNα/β activates not only the immune system but also many effector proteins for protein synthesis and cell proliferation [11].

The tyrosine kinase 2 (*Tyk2*) gene encodes a signal-transducing molecule associated with IFN receptor, IL-12 receptor, and IL-23 receptor, mediating interferon response, both Th1- and Th17-type immune reactions, and playing a major role in resistance to various infections including viruses, bacteria, and parasites [12,13]. In the context of the pathogenesis of viral diabetes, encephalomyocarditis virus strain D (EMC-D strain) has been well studied in rodents [3]. Recently, we reported that naturally occurring mutation of the *Tyk2* gene reduced *TYK2* gene expression in islets and EMC-D virus infection caused severe islet cell damage with impaired IFN-related response in mice with natural mutation of the *Tyk2 gene* [14]. Moreover, we investigated the role of genetic polymorphism of the *TYK2* gene in patients with type 1 diabetes including those with flu-like symptoms at the onset of diabetes [15]. We found that polymorphisms of the promoter region and exon 1 of the *TYK2* gene showed complete linkage disequilibrium and named the associated haplotype *TYK2* promoter variant (*TYK2PV*) (NCBI ClinVar ID: 440728: https://www.ncbi.nlm.nih.gov/clinvar/variation/440728/ (accessed on 15 December 2019). We also found that the prevalence of *TYK2PV* in patients with type 1 diabetes was higher than in healthy controls and that *TYK2PV* was associated with a significant risk of type 1 diabetes (odds ratio 2.4) and of type 1 diabetes with a flu-like syndrome at disease onset (odds ratio 3.6) independent of positivity or negativity for the GAD antibody. Interestingly, *TYK2PV* was also identified as a risk factor for type 2 diabetes (odds ratio 2.1). Taking these findings together, our previous study suggests that *TYK2PV* is associated with the pathogenesis of type 2 diabetes as well as type 1 diabetes, which might mediate virus-induced islet damage. Therefore, we hypothesized that insulin secretion in type 2 diabetes is associated with *TYK2PV*, which might reveal a unique and novel disease type of type 2 diabetes. In the current study, we thus focused on the insulin secretion in patients with type 2 diabetes and studied its relationship with *TYK2PV*.

## 2. Materials and Methods

### 2.1. Patients

We prospectively analyzed 1004 patients with type 2 diabetes who visited Saga University Hospital (Saga, Japan), Saiseikai Karatsu Hospital (Karatsu, Japan), Eguchi Hospital (Ogi, Japan), Takagi Hospital (Okawa, Japan), and Inuzuka Hospital (Kashima, Japan) between September 2016 and May 2018. The diagnosis of type 2 diabetes was made in accordance with the diagnostic criteria of Standards of Medical Care in Diabetes 2016 [16]. The patients with chronic kidney disease (serum creatinine of ≥ 1.0 mg/dL or estimated glomerular filtration rate (eGFR) of < 60 mL/min/1.73 m^2^) and/or taking medication including steroid agents, immunosuppressive agents, insulin injection, or sulfonyl urea were excluded because these could affect glucose metabolism and endogenous insulin secretion. Consequently, 172 patients were enrolled in this study. The study protocol was approved by the Clinical Research Ethics Review Committee of Saga University Hospital (approval number: 2016-04-11) and the individual institutions. The study was conducted in accordance with the principles of the 1975 Declaration of Helsinki, as revised in 2013. Written informed consent was obtained from all participants in this study.

### 2.2. Physical Examination and Serum Biochemical Measurements

Body mass and height were measured, and body mass index (BMI) was calculated as body mass (kg) divided by the square of height (m2). Information about comorbid diseases and medications was collected from the medical records by a physician. Venous blood samples were obtained after overnight fasting and used for measuring the counts of white blood cells and their subtypes, hemoglobin, platelets, creatinine (Cr), eGFR, aspartate aminotransferase (AST), alanine aminotransferase (ALT), fasting plasma glucose, glycated hemoglobin, insulin, high-density-lipoprotein cholesterol (HDL-C), low-density-lipoprotein cholesterol (LDL-C), and triglycerides (TG), using conventional laboratory techniques. Insulin resistance was evaluated using homeostasis model assessment of insulin resistance (HOMA-IR; fasting insulin (IU/mL) × fasting blood sugar (mg/dl)/405). eGFR was calculated using the following equation: 194 × Cr (mg/dL)^−1.094^  ×  age (year)^−0.287^ for men, and the values obtained for women were multiplied by 0.739 [17].

For evaluation of the ability to secrete insulin, homeostatic model assessment β-cell function (HOMA-β), fasting C-peptide, and C-peptide index (CPI) were examined [18]. HOMA-β was calculated using the following equation: fasting insulin × 360/([fasting blood sugar (mg/dl) − 63) [19]. CPI was calculated using the following equation: fasting C-peptide (ng/mL)/fasting blood sugar (mg/dl) × 100 [20].

### 2.3. DNA Extraction and Genotyping of TYK2PV

For the extraction of DNA from whole blood, an automatic nucleic acid extraction system (Quick Gene DNA whole blood kit S (Fujifilm, Cat. No. DB-S)) was used in accordance with the manufacturer’s protocol. The sequence of *TYK2PV* was in accordance with NCBI Reference Sequence: ClinVar ID: 440728. Two single-nucleotide polymorphisms (SNPs) were discriminated in our previous study: NC_000019.10:g.10381501A>T(GRCh38) (rs891696485) [15] and NC_000019.10:g.10381502C>T(GRCh38) (rs953883300). The genomic region containing the SNPs was amplified by PCR with a forward primer (5′-GCCGAGATCGCATTG-3′) and a reverse primer (5′-CAAAGACCGTCCTCTGACCC-3′). The PCR conditions were as follows: initial denaturation at 96°C for 2 min; then 40 cycles of 96 °C for 20 s, 66 °C for 20 s, and 72 °C for 20 s; followed by a final extension step at 72 °C for 2 min. The size of the PCR products was verified by agarose gel electrophoresis. After the PCR product was refined using the ExoSAP-IT (Thermo Fisher Science), the sequencing reaction was performed with a sequencing primer (5′-ACCCTTCTTCTGTGCCACAC-3′) in a Bio-Rad DNA Engine Dyad PTC-220 Peltier Thermal Cycler using ABI PRISM^®®^ BigDye^®®^ Terminator v3.1 Cycle Sequencing Kits (Applied Biosystems), following the protocols supplied by the manufacturer. The genotype was defined as wild type (AC/AC) or *TYK2PV* (AC/TT or TT/TT). The patients with *TYK2PV* (AC/TT or TT/TT) were defined as the TYK2PV group.

### 2.4. Statistical Analysis

Continuous variables are presented as the median and 25th to 75th percentile range. The chi-squared test or Fisher’s exact test was used to perform intergroup comparisons of categorical variables. The Wilcoxon rank sum test was used for intergroup comparisons of continuous variables. Spearman’s rank correlation coefficient was used for the correlation analysis. A logistic regression model was used to perform multivariate analysis for the factors associated with the ability to secrete insulin. Explanatory variables were those significant in the comparisons of the demographics and other characteristics between *TYK2PV* and the wild type in Table 1. Statistical significance was defined as *p* < 0.05. All analyses were performed using JMP^®®^ 14 (SAS Institute Inc., Cary, NC, USA).

## 3. Results

### 3.1. Characteristics of the Patients

**Figure 1:**
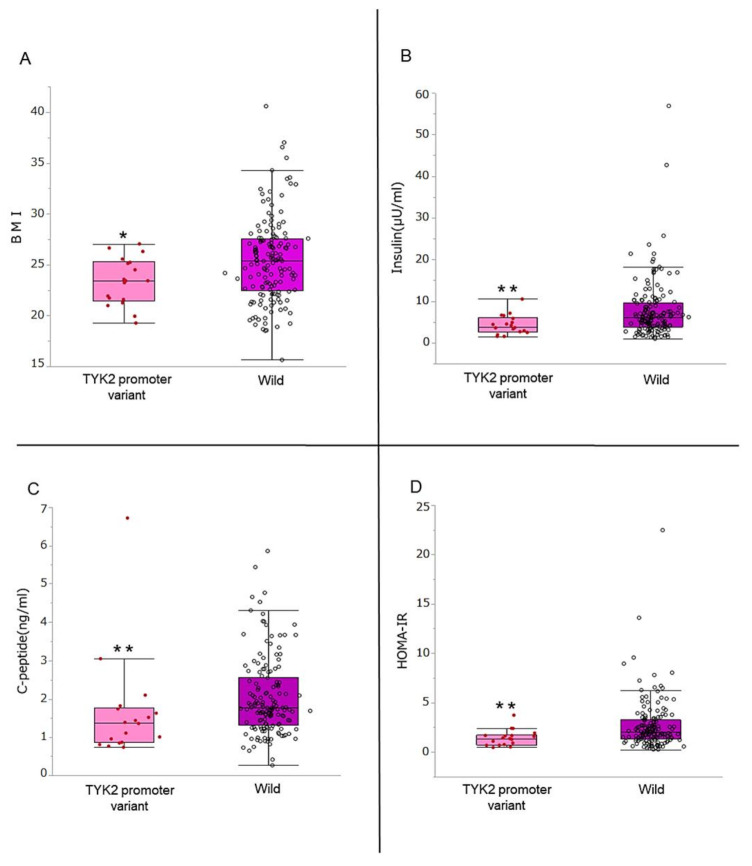
Association of *TYK2PV* with body mass index (BMI), insulin secretory ability, and insulin resistance. (**A**) BMI, (**B**) insulin secretion ability evaluated using fasting insulin, (**C**) C-peptide, and (**D**) insulin resistance (HOMA-IR) were compared between those with the *TYK2* promoter variant and the wild type. * *p* < 0.05 and ** *p* < 0.01.

The patients’ characteristics were summarized and compared between *TYK2PV* and the wild type (Table 1). In the 172 patients with type 2 diabetes, *TYK2PV* was identified in 18 patients (10.5%). The BMI of the patients with *TYK2PV* was significantly lower than that of the wild-type group (*p* = 0.025). There were no differences in age, gender, or disease duration. The prevalence of concomitant lifestyle-related diseases including hypertension, dyslipidemia, and hyperuricemia did not differ between the genotypes. There was also no difference in the frequency of prescribed medication for diabetes, nor in blood cell counts and leukocyte subtypes, renal function, liver enzymes, cholesterols, or triglyceride. Moreover, fasting plasma glucose and glycated hemoglobin did not differ between the genotypes. However, in the comparisons of insulin secretion ability, all evaluated parameters including fasting insulin (*p* = 0.007), HOMA-β (*p* = 0.021), C-peptide (*p* = 0.008), and C-peptide index (*p* = 0.016) were significantly lower in *TYK2PV* than in the wild type (also summarized in Figure 1). Insulin resistance of the *TYK2PV* group was also significantly lower than in the wild type. Finally, GAD antibody was negative in all patients in the *TYK2PV* and wild-type groups.

### 3.2. Comparison of BMI, Insulin Secretion Ability, and Insulin Resistance

Among the variables that significantly differed between the genotypes, the comparisons of BMI, fasting insulin, C-peptide, and HOMA-IR are displayed graphically in Figure 1. BMI of the patients with *TYK2PV* was significantly lower than in the wild type (23.4 vs. 25.4 kg/m^2^, *p* = 0.025) and there were no patients with BMI > 30 kg/m^2^ in the *TYK2PV* group (Figure 1A). Fasting insulin (3.9 vs. 6.2 μIU/mL, *p* = 0.007) (Figure 1B) and C-peptide (1.37 vs. 1.76 ng/mL, *p* = 0.008) (Figure 1C) were significantly lower in the patients with *TYK2PV* than in the wild type, as was HOMA-IR (Figure 1D).

### 3.3. Association of TYK2PV with the Ability to Secrete Insulin, Insulin Resistance, and BMI

**Figure 2:**
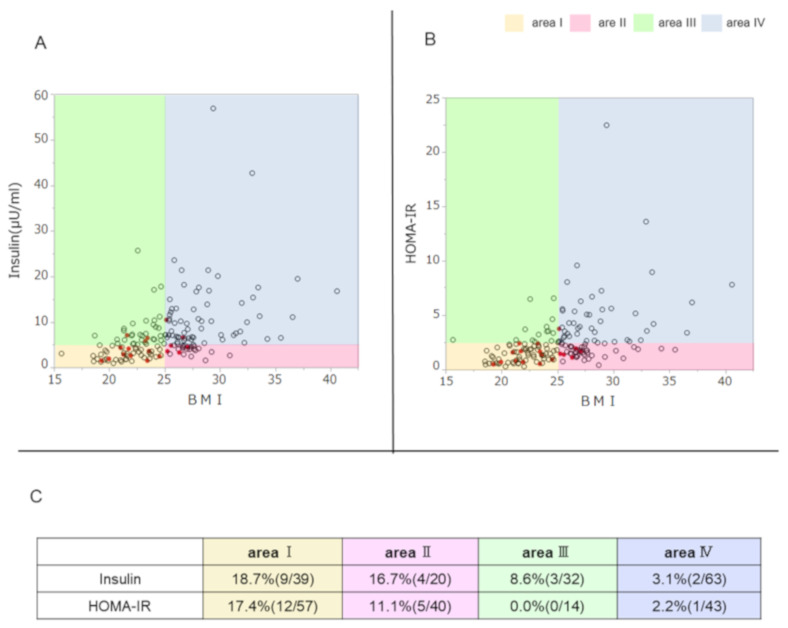
Association of *TYK2PV* with the ability to secrete insulin, insulin resistance, and BMI. *TYK2PV* is plotted with red circles and the wild type is plotted with open circles. The graph area was divided according to the BMI (25 kg/m^2^) and insulin secretion ability (**A**, insulin, 5 μU/mL) or insulin resistance (**B**, HOMA-IR, 2.5). Individual areas were defined as area I in yellow, area II in pink, area III in green, and area IV in blue. (**C**) Prevalence of *TYK2PV* in the individual areas is summarized.

In the graphs showing the correlation of BMI and insulin secretion ability or insulin resistance, *TYK2PV* was plotted to visualize the association with these parameters (Figure 2). There was a positive correlation between fasting insulin and BMI (ρ = 0.508, *p* < 0.001) (Figure 2A). According to the fasting insulin level (5 μU/m) and BMI (25 kg/m^2^), the graph area was divided into four segments: area I (low insulin secretion ability and low BMI), area II (low insulin secretion ability and high BMI), area III (high insulin secretion ability and low BMI), and area IV (high insulin secretion ability and high BMI). The prevalence of *TYK2PV* was highest in area I, followed by area II, and lowest in area IV, with a significant difference (*p* = 0.021) (Figure 2C). In accordance with insulin resistance, HOMA-IR positively correlated with BMI (ρ = 0.486, *p* < 0.001) (Figure 2B). According to HOMA-IR (2.5) and BMI (25 kg/m^2^), the graph area was also again divided into four segments: area I (low insulin resistance and low BMI), area II (low insulin resistance and high BMI), area III (high insulin resistance and low BMI), and area IV (high insulin resistance and high BMI). The prevalence of *TYK2PV* was highest in area I, followed by area II, and lowest in area III, with a significant difference (*p* = 0.039) (Figure 2C).

### 3.4. Effect of TYK2PV on Insulin Secretion Ability and Insulin Resistance: Multivariate Analysis

**Figure 3:**
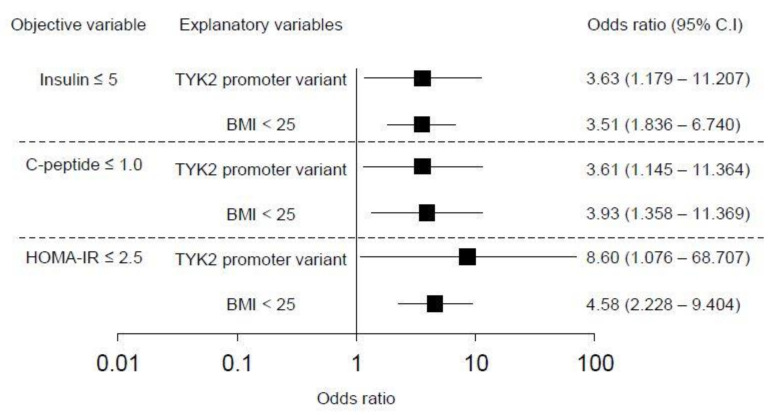
Multivariate analysis of the associations with insulin secretion ability and insulin resistance. Factors associated with fasting insulin ≤ 5 μU/mL, C-peptide ≤ 1.0, and HOMA-IR ≤ 2.5 were tested. Black box and error bar represent odds ratio and 95% confidence interval (95% CI), respectively.

In the overall patients, multivariate analysis was performed to test whether *TYK2PV* is independently associated with insulin secretion ability and insulin resistance. *TYK2PV* was significantly associated with fasting insulin ≤ 5 μIU/mL (odds ratio 3.63, *p* = 0.025) and C-peptide ≤ 1.0 ng/mL (odds ratio 3.61, *p* = 0.028) independent of BMI < 25 kg/m^2^ (Figure 3). *TYK2PV* was also associated with lower insulin resistance; *TYK2PV* was associated with HOMA-IR ≤ 2.5 (odds ratio 8.60, *p* = 0.042) independent of BMI < 25 kg/m^2^ (Figure 3).

## 4. Discussion and Conclusions

Although type 2 diabetes and its pathogenesis are mainly linked to lifestyle-related factors including obesity and metabolic syndrome, the current study demonstrates that genetic background, in the form of *TYK2PV*, is associated with impaired insulin secretion ability of type 2 diabetes patients. Our data suggest that viral infectious disease (VID) is potentially involved in the pathogenesis of type 2 diabetes concomitant with other known risk factors for the development of this condition.

*TYK2PV* is a putative susceptibility gene of VID based on mouse and human studies; naturally occurring mutation of the *TYK2* gene in VID-susceptible SJL mice was shown to be responsible for such susceptibility. Meanwhile, in humans, those with *TYK2PV* presented an odds ratio of 2.4, which is most highly related to anti-GAD autoantibody negativity and flu-like syndrome associated with the onset of type 1 diabetes [14,15]. In contrast, as presented in the current paper, patients with type 2 diabetes with *TYK2PV* did not have apparent clinical manifestations of infection at least at the onset, suggesting that the role of infection may be latent or a preceding event, rather than directly leading to disease onset. Viral infection causes a wide range of disease outcomes dependent on the pathogenicity of the virus, challenged viral dose, route of infection, and host resistance factors, most importantly immunoprotective mechanisms. Accordingly, viral infection even by a single virus causes a vast range of clinical phenotypes, including fulminant, acute, mild, chronic, and persistent types, as well as a subclinical symptomless condition. Thus, viral infection is a consistent risk for the development of type 2 diabetes, although this has not been well recognized.** Richardson et al. reported that enterovirus-driven pancreatic inflammation stained with the enteroviral capsid antigen vp1 was identified in the 40% of type 2 diabetes patients, which was higher than the healthy non-diabetic control (13%) [21]. On the other hand, Gkrania-Klotsas et al. reported that the prevalence of Coxsackievirus B neutralizing antibodies was not different between the type 2 diabetes patients and the subjects without diabetes [22]. In the type 2 diabetes patients of sub-Saharan African, positive human herpesvirus 8 DNA associated with lower insulin secretion and lower BMI comparing with the type 2 diabetes patients without human herpesvirus 8 infection [23]. Similarly, hepatitis C virus infection associates with the lower insulin secretion in the type 2 diabetes patients [24]. Whereas the accumulating evidences directly or indirectly imply the association between the virus infection and type 2 diabetes, to date, it has not been confirmed. The damage of pancreatic β cell by the virus infection would be a part of pathogenesis of type 2 diabetes and virus infection might be one of the risk factors.

**Figure 4:**
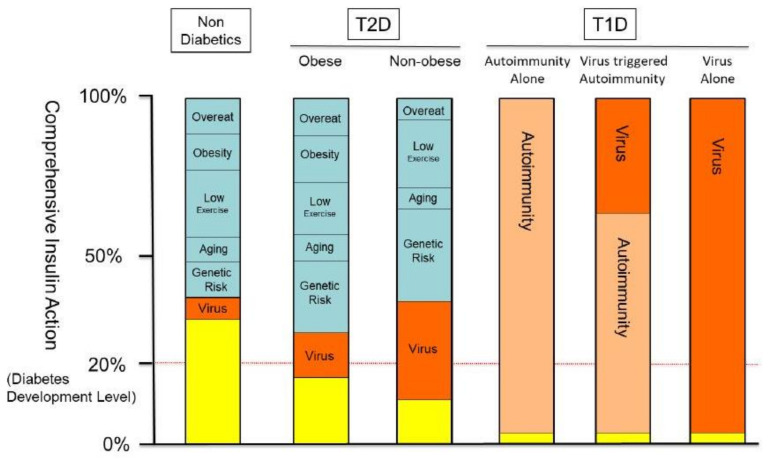
The role of viral infection in comprehensive insulin activity in type 1 and type 2 diabetes. The pathogenesis of diabetes is a result of impaired insulin activity. Residual insulin activity (yellow) can be defined by subtracting the impairment of insulin activity due to insulin secretion and/or insulin resistance, caused by overeating, obesity, low exercise, aging, genetic risk, and viral infection (orange). It has been elucidated that an autoimmune reaction triggered by viral infection, as well as the direct disruption caused by viral infection, impairs the insulin secretion of β cells and is associated with the disease type of type 1 diabetes. As demonstrated in the current study, viral infection is also associated with type 2 diabetes. The effect of viral infection on the comprehensive activity of insulin is more prominent in nonobese patients than in obese ones in type 2 diabetes.

In type 2 diabetes, risk factors are variable and depend on the individual’s condition, including genetic risks, obesity, overeating, low exercise, aging, biological activity of β cells, and, as shown here, viral infection (Figure 4). Although genome-wide study is a powerful approach to identify the risk factors of type 2 diabetes and has revealed more than 70 SNPs associated with type 2 diabetes globally, irrespective of ethnic background [25,26], the biological significance of those SNPs has not yet been clarified and the collective contribution of those SNPs has yet to be reported [27]. SNPs related to the ability to secrete insulin have also been investigated in type 2 diabetes; however, the effects of the variants were shown to be modest [28,29]. In contrast, as shown by our previous study and the current work, *TYK2PV* demonstrated a consistent effect: increasing the risk of type 2 diabetes by 2.1-fold [14] and the risk of impaired insulin secretion ability by 3.2–3.5-fold.

In the current study, the prevalence of *TYK2PV* was higher in patients with lower BMI. A previous epidemiological study suggested that the average BMI of Japanese patients with type 2 diabetes is lower than that in Caucasians [30,31], although the mechanism associated with the difference in BMI remains unclear. Even in the early phase of type 2 diabetes, insulin secretion ability can be impaired in East Asians [32,33]. Indeed, our results showed that lower BMI was an independent risk factor for impaired insulin secretion. Moreover, insulin resistance was not more severe in the *TYK2PV* group than in the wild-type group. Not a few patients with *TYK2PV* who tend not to be obese and to have less insulin resistance have been categorized with type 2 diabetes.

In terms of the clinical implications of this study, *TYK2PV* could affect the therapeutic strategy for type 2 diabetes. Basically, insulin resistance is a common pathogenic factor in type 2 diabetes and targeted by pharmacological therapy including metformin and sodium glucose cotransporter inhibitors [34,35]. However, patients with *TYK2PV* diagnosed with type 2 diabetes might not follow this strategy. To protect and recover β-cell function, recent evidence has encouraged the use of insulin treatment for type 1 diabetes patients retaining the ability to secrete insulin and type 2 diabetes patients with an early stage of insulin secretion impairment, similar to the treatment for typical type 1 diabetes [36]. In type 2 diabetes, poor glycemic control with antidiabetic agents is considered to be an indication of insulin treatment. Severe β-cell dysfunction caused by hyperglycemia-induced glucotoxicity aggravates basal insulin secretion [37]. Moreover, α-cell dysfunction could be concomitant with β-cell dysfunction and paradoxically lead to the secretion of glucagon under hyperglycemic conditions [38]. According to large clinical trials, insulin treatment rather than sulfonyl urea ameliorated β-cell dysfunction and prevented the progression of diabetic macro- and microvascular complications [36,39,40]. According to our previous study and the current work, approximately 10% of type 2 diabetes patients have *TYK2PV* [15]. Moreover, these studies excluded patients treated with insulin, suggesting that the prevalence of *TYK2PV* could be much higher than we identified. Type 2 diabetes patients with *TYK2PV* should be carefully assessed to determine whether the diabetes has reached the insulin-dependent state and should be treated appropriately with insulin if necessary.

There are several limitations to the current study. Because our study design was cross-sectional, it remained unclear whether *TYK2PV* was associated with the onset of type 2 diabetes. A longitudinal study design including healthy subjects with *TYK2PV* should make this more evident. All insulin secretion parameters evaluated in this study were evaluated under a fasting state. Therefore, the association between *TYK2PV* and additional insulin secretion remains unclear. However, it was reported that there was a significant positive correlation between the fasting insulin secretion ability and additional insulin secretion evaluated by hyperglycemic clamp study [41]. A preventive enteroviral vaccine against VID is now under development [42,43,44]. Based on the current study, this vaccine might contribute not only to preventing T1D, but also to reducing the risk of type 2 diabetes associated with enteroviral infection. Moreover, the evaluation of *TYK2PV* could contribute to determining which patients should be vaccinated as a priority.

In conclusion, *TYK2PV* is related to impaired insulin secretion and low insulin resistance in type 2 diabetes. It is important to recognize the different disease phenotype of such cases compared with general type 2 diabetes and provide appropriate care and preventive measures.

## Figures and Tables

**Table 1:** Baseline characteristics of the patients.

Characteristic	Overall (*n* = 172)	*TYK2PV* (*n* = 18)	Wild Type (*n* = 154)	*p*-Value *
Age, yr.	65.5 (57–71)	62.5 (52.7–73.2)	66 (57–71)	0.783
Female sex, no. (%)	64 (37.2)	4 (22.2)	60 (38.9)	0.164
Duration of diabetes, yr.	9 (4–15.5)	10.0 (6.5–15.0)	9 (4.0–15.7)	0.93
Body weight, kg	63.6 (56.2–71.9)	61.4 (54.5–70.4)	64 (56.2–72.9)	0.457
BMI, kg/m^2^	25.2 (22.3–27.4)	23.4 (21.5–25.3)	25.4 (22.5–27.6)	0.025
Hypertension, no. (%)	82 (47.6)	5 (27)	77 (50.0)	0.074
Dyslipidemia, no. (%)	89 (51.7)	8 (44.4)	81 (52.5)	0.512
Hyperuricemia, no. (%)	10 (5.8)	2 (11.1)	8 (5.2)	0.310
Dietetic therapy, no. (%)	17 (10.0)	3 (16.6)	14 (9.0)	0.318
α-Glucosidase inhibitors, no. (%)	30 (17.4)	3 (16.6)	27 (17.5)	0.927
Meglitinides, no. (%)	7 (4.1)	1 (5.5)	7 (4.5)	0.355
Thiazolidinediones, no. (%)	9 (5.2)	6 (33.3)	8 (5.2)	0.948
Metformin, no. (%)	76 (44.1)	6 (33.3)	70 (45.4)	0.327
DPP-4inhibitors, no. (%)	106 (61.6)	11 (61.1)	95 (61.0)	0.962
GLP-1Ra, no. (%)	7 (4.0)	2 (11.1)	5 (3.2)	0.110
SGLT-2 inhibitor, no. (%)	32 (19.1)	2 (11.1)	31 (20.1)	0.357
White blood cell, /μl	6080 (4940–7100)	5600 (4790–6350)	6100 (4993–7210)	0.271
Hemoglobin, g/dl	14.4 (13.6–15.5)	14.7 (13.95–15.85)	14.4 (13.5–15.5)	0.320
Platelet, x10^4^/μl	21.8 (18.1–26.0)	23.9 (18.4–25.8)	21.6 (17.8–26.0)	0.545
Neutrophils, %	58.1 (52.1–64.2)	60.0 (54.5–65.0)	58.1 (52.0–64.2)	0.672
Lymphocytes, %	31.8 (26.3–38.5)	32.1 (25.7–36.7)	31.8 (26.5–38.7)	0.583
Monocytes, %	5.3 (4.4–6.1)	5.0 (4.35–5.85)	5.4 (4.4–6.2)	0.456
Eosinophils, %	2.4 (1.6–3.9)	2.0 (1.5–4.2)	2.4 (1.6–3.6)	0.869
Basophils, %	0.5 (0.4–0.9)	0.5 (0.2–0.7)	0.5 (0.4–0.9)	0.246
Cr, mg/dl	0.74 (0.61–0.84)	0.76 (0.67–0.85)	0.72 (0.60–0.84)	0.230
eGFR, ml/min/1.73m^2^	77.0 (67.0–88.2)	79.6 (68.6–90.5)	76.9 (67.0–87.5)	0.779
AST, U/l	22 (18–27)	24.0 (15.7–27.2)	22.0 (18.0–27.3)	0.580
ALT, U/l	22 (16–33)	21.5 (14.7–28.2)	22.5 (16–33.3)	0.604
HDL, mmol/L	1.48 (1.24–1.68)	1.42 (1.22–1.67)	1.48 (1.24–1.70)	0.491
LDL, mmol/L	2.85 (2.25–3.39)	2.67 (2.21–3.16)	2.87 (2.25–3.42)	0.302
Triglyceride, mmol/L	1.23 (0.88–1.67)	1.03 (0.75–1.27)	1.28 (0.92–1.69)	0.069
Fasting plasma glucose, mg/dl	137 (117–153)	139 (116–149)	136 (116–154)	0.924
HbA1c, mmol/mol (%)	52(7.0) {46(6.4)-59(7.6)}	50(6.8) {46(6.4)-57(7.4)}	52(7.0) {46(6.4)-59(7.6)}	0.741
Insulin, μU/ml	6.0 (3.6–8.9)	3.9 (2.6–6.0)	6.2 (3.9–9.7)	0.007
HOMA-β	29.9 (17.3–47.1)	19.1 (12.3–34.7)	30.5 (18.7–48.3)	0.021
HOMA-IR	1.91 (1.23–3.06)	1.39 (0.73–1.73)	2.05 (1.28–3.30)	0.006
C-peptide, ng/ml	1.72 (1.26–2.44)	1.37 (0.86–1.76)	1.76 (1.32–2.55)	0.008
C-peptide index	1.28 (0.92–1.74)	0.90 (0.70–1.38)	1.32 (0.96–1.77)	0.016
Anti-GAD-Ab positive, no. (%)	0 (0)	0 (0)	0 (0)	-

or number (%). * The *p*-value refers to the difference between *TYK2PV* and wild type. *TYK2PV*, *TYK2* promoter variant; DPP4 inhibitor, dipeptidyl peptidase-4 inhibitor; GLP-1Ra, glucagon-like peptide-1 receptor agonist; SGLT2 inhibitor, sodium glucose cotransporter 2 inhibitor; HOMA-β, homeostatic model assessment of β-cell function; HOMA-IR, homeostasis model assessment of insulin resistance; anti-GAD-Ab, anti-GAD antibody.

## Data Availability

The data presented in this study are available on request from the corresponding author. The data are not publicly available due to privacy.

## Prior Presentation

Parts of this study were presented in poster from at the 79th Scientific Sessions of the American Diabetes Association, San Francisco, CA, 7–11 June 2019.
